# Cross-Reactivity Using Chimeric Trypanosoma cruzi Antigens: Diagnostic Performance in Settings Where Chagas Disease and American Cutaneous or Visceral Leishmaniasis Are Coendemic

**DOI:** 10.1128/JCM.00762-19

**Published:** 2019-07-26

**Authors:** Ramona Tavares Daltro, Leonardo Maia Leony, Natália Erdens Maron Freitas, Ângelo Antônio Oliveira Silva, Emily Ferreira Santos, Rodrigo Pimenta Del-Rei, Maria Edileuza Felinto Brito, Sinval Pinto Brandão-Filho, Yara Miranda Gomes, Marcelo Sousa Silva, Silvia Tavares Donato, Selma Maria Bezerra Jeronimo, Gloria Regina de Góis Monteiro, Lucas Pedreira Carvalho, Andréa Santos Magalhães, Nilson Ivo Tonin Zanchin, Paola Alejandra Fiorani Celedon, Fred Luciano Neves Santos

**Affiliations:** a Gonçalo Moniz Institute, Oswaldo Cruz Foundation, Salvador, Bahia, Brazil; b Faculty of Technology and Sciences of Bahia, Salvador, Bahia, Brazil; c Aggeu Magalhães Institute, Oswaldo Cruz Foundation, Recife, Pernambuco, Brazil; d Federal University of Rio Grande do Norte, Natal, Rio Grande do Norte, Brazil; e Institute of Hygiene and Tropical Medicine, Lisbon, Portugal; f Federal University of Bahia, Salvador, Bahia, Brazil; g Carlos Chagas Institute, Oswaldo Cruz Foundation, Curitiba, Paraná, Brazil; h Molecular Biology Institute of Paraná, Curitiba, Paraná, Brazil; Cepheid

**Keywords:** American cutaneous leishmaniasis, Chagas disease, cross-reactivity, recombinant chimeric antigens, visceral leishmaniasis

## Abstract

Chimeric T. cruzi antigens have been proposed as a diagnostic tool for chronic Chagas disease (CD) in both settings where Chagas disease is endemic and those where it is not endemic. Antibody response varies in accordance to each T. cruzi strain, presenting challenges to the use of antigens lacking demonstrated cross-reactivity with Leishmania spp. Our group expressed four chimeric proteins (IBMP-8.1, IBMP-8.2, IBMP-8.3, and IBMP-8.4) and previously assessed their diagnostic performance to determine cross-reactivity with *Leishmania* spp. Here, we validated our findings using serum samples from different Brazilian geographic areas reporting endemic Chagas disease, endemic visceral or American cutaneous leishmaniasis (ACL), or both. Overall, 829 serum samples were evaluated using commercial and IBMP enzyme-linked immunosorbent assays. Due to the absence of a reference assay to diagnosis CD, latent class analysis (LCA) was performed through the use of a statistical model. The incidence of cross-reactivity for ACL-positive samples varied from 0.35% (IBMP-8.3) to 0.70% (IBMP-8.1 and IBMP-8.2). Regarding visceral leishmaniasis (VL)-positive samples, the IBMP-8.2 and IBMP-8.3 antigens cross-reacted with six (3.49%) and with only one sample (0.58%), respectively. No cross-reactivity with either ACL or VL was observed for the IBMP-8.4 antigen. Similarly, no cross-reactions were found when VL-positive samples were assayed with IBMP-8.1. The agreement among the results obtained using IBMP antigens ranged from 97.3% for IBMP-8.2 and 99% for IBMP-8.1 and IBMP-8.3 to 100% for IBMP-8.4, demonstrating almost perfect agreement with LCA. Accordingly, in light of the negligible cross-reactivity with both ACL and VL, we suggest the use of IBMP antigens in regions where T. cruzi and *Leishmania* spp. are coendemic.

## Abstract (precis)

Chimeric T. cruzi antigens have been proposed as a diagnostic tool for chronic Chagas disease (CD) in both settings where Chagas disease is endemic and those where it is not endemic. Antibody response varies in accordance to each T. cruzi strain, presenting challenges to the use of antigens lacking demonstrated cross-reactivity with Leishmania spp.

## INTRODUCTION

Human Chagas disease (CD) is considered the most critical life-threatening neglected tropical condition in 21 Latin American countries, affecting an estimated 6 to 7 million people and placing about 70 million individuals at risk of infection (1). CD is caused by the flagellated protozoan parasite Trypanosoma cruzi, which can be transmitted through the contaminated feces of blood-sucking triatomine bugs, by blood transfusion, from mother to child during pregnancy, during organ transplantation, or via oral transmission through ingestion of contaminated food. Since the late 1990s, human migratory flows have contributed to the dissemination of disease beyond the borders of Latin America, especially in Europe, Oceania, and North American countries (2–4).

The laboratory diagnosis of CD depends on the stage of disease. The acute phase is generally asymptomatic and lasts for about 2 months. Due to the high levels of parasitemia in this early stage, diagnosis is based on the visualization of trypomastigotes by staining thick and thin blood smears, considered the gold standard for CD diagnosis. After the acute phase, a period of lifelong chronic infection follows. Characterized by no or low/intermittent parasitemia and high levels of specific anti-T. cruzi antibodies (IgG), diagnosis during this stage necessitates the use of antigen-antibody detection methods, including indirect immunofluorescence (IIF), rapid diagnostic tests (RDTs), indirect hemagglutination (IHA), enzyme-linked immunosorbent assay (ELISA) and, more recently, electrochemiluminescence. Given the lack of an accurate standard for the serological diagnosis of chronic T. cruzi in infected individuals, the Pan American Health Organization (PAHO) and the WHO conventionally advise the use of two serological assays based on different antigens (e.g., whole parasite lysate and recombinant antigens) and/or methodologies (e.g., ELISA and IIF, or ELISA and RDTs) concomitantly to achieve an accurate diagnosis (5, 6). Currently, no standardized approach has been adopted, and testing algorithms vary according to the application (blood/organ donor screening or diagnosis) and location (settings where disease is endemic or nonendemic) in question, further highlighting the necessity to develop a highly accurate diagnostic tool to be employed as a single assay, regardless of endemicity or diagnostic setting (7–10).

Due to its simplicity, ELISA, which is the most widely employed assay to diagnose T. cruzi infection, enables the possibility of simultaneously diagnosing many individuals in a single assay, as well as the ability for automation (11). The performance of ELISAs depends on the antigenic matrix employed to detect anti-T. cruzi antibodies (12, 13). Conventional tests make use of either fractionated T. cruzi lysates or whole-cell epimastigote homogenates, resulting in a complex antigenic mixture of unknown and variable composition. Despite the high sensitivity demonstrated by these tests, several drawbacks have been described, such as cross-reactivity with *Leishmania* spp. and Trypanosoma rangeli, which lead to difficulties in protocol standardization, as well as to low specificity (13–16). Unconventional ELISAs based on recombinant antigens attempt to circumvent interference from other components present when antigens are extracted from whole parasites (14). Despite the increased specificity offered by recombinant antigens, reports of cross-reactivity with *Leishmania* spp. have persisted (13, 16–19). As a result, commercial tests employing recombinant antigenic matrices have been known to lead to false-positive results in *Leishmania*-exposed individuals, primarily in settings in which these infections occur concomitantly—a growing problem since areas where each pathogen is endemic have overlapped in Brazil and elsewhere (16, 17, 20).

More recently, chimeric T. cruzi antigens, composed of conserved and repetitive amino acid fragments from several proteins of this parasite, have been proposed as a suitable tool for the diagnosis of chronic Chagas disease in both settings where it is endemic and nonendemic (20–24). In light of this scenario, our group expressed four chimeric proteins (IBMP-8.1, IBMP-8.2, IBMP-8.3, and IBMP-8.4) and assessed their diagnostic performance in the detection of specific anti-T. cruzi antibodies under ELISA and liquid microarray assays (20, 25, 26). Initially, the degree of cross-reactivity with *Leishmania* spp. has been reportedly lower or absent under both ELISA (20) and liquid microarray platforms (25) in comparison to commercial tests (13). However, considering the limited number of samples analyzed and the lack of definitive clinical-form leishmaniasis characterization in these initial studies, we endeavored to conduct a more in-depth assessment of IBMP chimeric antigen cross-reactivity using a large number of well-established *Leishmania*-positive sera from different areas of Brazil where it is endemic, in which overlapping endemicity for both pathogens has been demonstrated.

## MATERIALS AND METHODS

### Ethical statement.

This study received approval from the IRB for Human Research of the Gonçalo Moniz Institute (IGM/Fiocruz-BA), Salvador-Bahia, Brazil (protocol no. 67809417.0.0000.0040), from the IRB of the Aggeu Magalhães Institute (IAM/Fiocruz-PE), Recife-Pernambuco, Brazil (protocol no. 15812213.8.0000.5190), and the IRB of the Federal University of Rio Grande do Norte (UFRN; protocol no. 12584513.1.0000.5537). Samples were obtained from the serum banks of collaborating institutions and incorporated into the biorepository of the Advanced Public Health Laboratory (LASP/IGM). In order to maintain patient information confidentiality, sera were coded so that the investigators were blind to research participants’ records, thereby avoiding the need for verbal or written consent.

### Recombinant chimeric protein preparation.

Four previously described T. cruzi chimeric proteins (20, 26) were cloned into the pET28a vector and expressed in Escherichia coli BL21-Star. E. coli lysates were prepared, and His-tagged chimeric antigens were purified by affinity and ion-exchange chromatography. The concentrations of purified chimeric antigens were determined by fluorometric quantification (Qubit 2.0, Invitrogen Technologies, Carlsbad, CA, USA) following the manufacturer’s protocol, and all recombinant proteins were stored at −20°C until employed for ELISA evaluation.

### Sampling.

**FIG 1:**
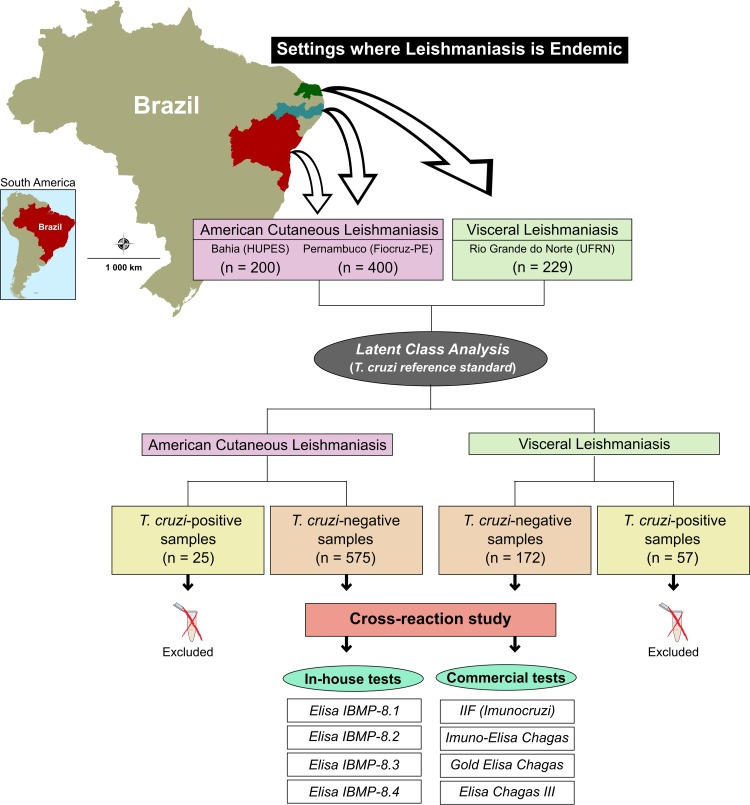
Study design in accordance with the Standards for Reporting of Diagnostic Accuracy Studies (STARD) guidelines. Note that with respect to the IIF or Imuno-ELISA Chagas tests, results were not available for all ACL and VL samples due to the unavailability of these previously commercially available test kits. Public domain digital maps were obtained from the Brazilian Institute of Geography and Statistics (IBGE) cartographic database in shapefile format (.shp), which was subsequently reformatted and analyzed using TerraView version 4.2, open source software freely available from the National Institute for Space Research (www.dpi.inpe.br/terraview).

Anonymized human serum samples were provided by the respective laboratories of the Leishmaniasis Reference Service of the IAM/Fiocruz-PE, the Professor Edgard Santos University Hospital at the Federal University of Bahia (HUPES/UFBA), and the Federal University of Rio Grande do Norte (UFRN). Sampling size was calculated using a negative binomial distribution, assuming that 10% of the samples would possibly cross-react with leishmaniasis. Accordingly, a minimum sample size was determined, comprising 200 sera positive for American cutaneous leishmaniasis (ACL) and 200 sera presenting reactivity for visceral leishmaniasis (VL). In total, 829 anonymized human sera were assayed, including 600 samples characterized as positive for ACL (*n* = 400 from LRS/Fiocruz-PE; *n* = 200 from HUPES/UFBA) and 229 as positive for VL (UFRN). All samples were collected from areas of endemic leishmaniasis in states located in northeastern Brazil, Bahia, Pernambuco, and Rio Grande do Norte (Fig. 1). In addition to clinical evaluations, ACL diagnosis was also based on an association of several laboratory tests, as well as on epidemiological conditions (27–29). With regard to VL, samples were obtained from individuals with a clinical suspicion of VL who were hospitalized at an infectious disease reference unit. All patients presented fever, weight loss, and hepatosplenomegaly, and the definitive diagnosis of VL included hematological analysis indicating anemia, leukopenia and thrombocytopenia. Confirmation entailed the observation of parasites under direct examination and/or in cultures of bone marrow aspirate, as well as the detection of anti-*Leishmania* antibodies by serological testing (30).

### Study design.

All serum samples were simultaneously evaluated for cross-reactivity with Chagas antigens using commercial and in-house serological assays. Two commercial Chagas disease-specific enzyme-linked immunoassays tests were employed to analyze all 829 samples with previous positivity for leishmaniasis, Gold ELISA Chagas (REM, São Paulo, Brazil), which uses both recombinant antigens and purified lysates from Brazilian strains of T. cruzi epimastigotes, and ELISA Chagas III (BIOSChile, Ingeniería Genética S.A., Santiago, Chile), which uses whole extracts of T. cruzi strains Mn and Tulahuen as antigens. Moreover, the 400 ACL-positive samples from IAM/Fiocruz-PE were additionally assayed with the Imuno-ELISA Chagas kit (Wama Diagnóstica, São Paulo, Brazil), which employs recombinant antigens and indirect immunofluorescence (IIF) (Imunocruzi; bioMérieux, São Paulo, Brazil). All commercial ELISA testing was conducted in accordance with each respective manufacturer’s protocols. The Imuno-ELISA Chagas and Imunocruzi IIF tests were used to evaluate just 400 ACL-positive samples obtained from IAM/Fiocruz-PE; as production of these kits was discontinued, it was impossible to submit the entire sample to these specific tests. For IIF, glass slides coated with antigens from T. cruzi Y epimastigotes (Imunocruzi; bioMérieux, Brazil) were used. Sera were diluted at 1:40 in 0.05 M phosphate-buffered saline (PBS; pH 7.4). The overlaid antigens were incubated in a moist chamber for 30 min at 37°C and then washed three times with PBS. The antibody-antigen complex was overlaid with fluorescein isothiocyanate-conjugated goat anti-human IgG (Fluoline G; bioMérieux, Brazil) diluted 1:200 in 0.001% Evans blue in PBS solution. Incubation and washes were then repeated as described above. The slides were mounted in buffered glycerol and read using a Leica DMLS Binocular Microscope (Leica, Germany) at ×400 magnification. Positive results were visualized by green fluorescence, whereas no fluorescence indicated negative results.

Cross-reactivity with anti-*Leishmania* spp. specific antibodies was further evaluated using four IBMP antigens in an ELISA diagnostic platform (IBMP-ELISA). These assays were performed on 96-well transparent flat-bottom microplates (Nunc, Roskilde, Denmark) coated with one of the IBMP chimeric antigens at 12.5 ng (IBMP-8.2) or 25 ng per well (IBMP-8.1, IBMP-8.3, and IBMP-8.4) in carbonate buffer (0.05 M carbonate-bicarbonate, pH 9.6). The microplates were incubated with a synthetic blocking buffer (WellChampion; Ken-Em-Tec Diagnostics A/S, Taastrup, Denmark) in accordance with the manufacturer’s instructions. Serum samples were diluted at 1:100 in 0.05 M phosphate-buffered saline (PBS; pH 7.4), then loaded on the coated microplates and incubated at 37°C for 60 min. Subsequently, all wells were washed with PBS-0.05% Tween 20 (PBS-T; pH 7.4) and incubated at 37°C for 30 min with horseradish peroxidase (HRP)-conjugated goat anti-human IgG (Bio-Manguinhos/Fiocruz-RJ, Brazil) diluted 1:40,000 in PBS. After three washing cycles, 100 μl of a tetramethyl-benzidine chromogen (Kem-En-Tec, Taastrup, Denmark) was added, followed by incubation for 10 min at room temperature (RT) in the dark. Colorimetric reactions were interrupted by adding 50 μl of H_2_SO_4_ solution at 0.3 M and read in a microplate spectrophotometer (SpectraMax 340PC, San Jose, CA, USA) at an absorbance wavelength of 450 nm. Fig. 1 describes the study design according to the Standards for the Reporting of Diagnostic Accuracy Studies (STARD) guidelines (31).

### The use of latent class analysis as reference test.

Due to the absence of a reference assay for the diagnosis of CD, latent class analysis (LCA) was used as a reference standard and carried out employing a previously described and validated statistical model (32). In order to characterize the latent variable capable of correctly diagnosing T. cruzi infection, four indicators representing IBMP-8.1, IBMP-8.2, IBMP-8.3, and IBMP-8.4 chimeric antigens were established. Thus, latent class response patterns defined a given sample as T. cruzi positive if it presented positive results under at least two different chimeric-based assays (*a posteriori* probability varied from 87.9% to 100%). On the contrary, if a negative result was returned from all four chimeric antigens, or if positivity was seen from not more than one, then a given sample was considered negative for T. cruzi (*a posteriori* probability varied from 0 to 0.8%).

### Statistical data analysis.

GraphPad Prism software (version 7; San Diego, CA, USA) was employed for statistical analysis. Descriptive statistics are shown as geometric means plus or minus the 95% confidence interval (95% CI). A 5% level of significance was adopted for all statistical testing (*P* value < 0.05). In order to determine relevant cutoff (CO) values for the IBMP antigens, 10 T. cruzi-positive and 10 T. cruzi-negative samples were assayed in all microplates in parallel. These samples had been previously characterized as T. cruzi positive or negative based on two serological tests, in accordance with the World Health Organization’s diagnostic consensus (6). T. cruzi-positive samples were kindly provided by the Chagas Disease Reference Laboratory (Aggeu Magalhães Institute, Fiocruz-PE, Brazil), while negative samples were obtained from the Pernambuco Hematology and Hemotherapy Foundation (Hemope Foundation, Pernambuco, Brazil). CO values were established by constructing receiver operating characteristic (ROC) curves. All results from commercial and in-house (IBMP) ELISAs were measured by optical density (OD), with corresponding reactivity index (RI) values calculated as OD divided by CO. Results were interpreted as follows: negative (RI < 0.90), gray zone (0.90 ≥ RI ≤ 1.10), and cross-reactive (RI > 1.10). The strength of agreement between ELISA, using IBMP or commercial assays, and LCA results was assessed using Cohen’s Kappa coefficient (κ) (33), interpreted as follows: perfect (κ = 1.0), almost perfect (1.0 < κ > 0.80), substantial (0.80 ≤ κ > 0.60), moderate (0.60 ≤ κ > 0.40), fair (0.40 ≤ κ > 0.20), slight (0.20 ≤ κ > 0), or poor (κ = 0).

## RESULTS

**FIG 2:**
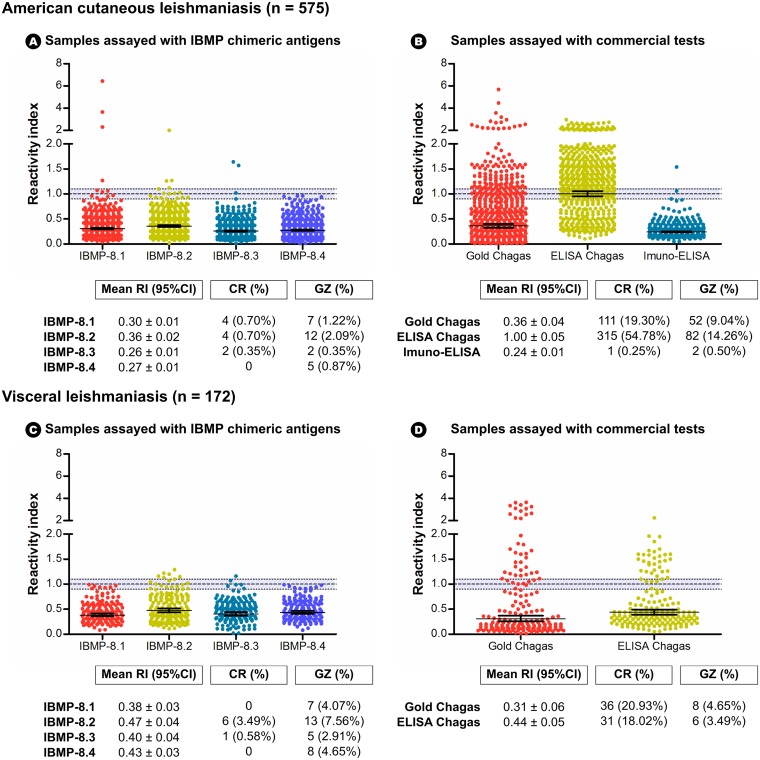
Reactivity index values from American cutaneous and visceral leishmaniasis-positive serum samples assayed with four IBMP chimeric antigens and commercial tests. RI = 1.0, cutoff; RI = 1.0% ± 10% (shaded area), gray zone. Horizontal lines for each group of results: geometric means (±95% CI). CR, cross-reactivity; GZ, gray zone; RI, reactivity index. Gold Chagas, Gold ELISA Chagas (Rem, Brazil); ELISA Chagas, ELISA Chagas III (BIOSChile, Ingeniería Genética S.A. Chile); Imuno-ELISA, Imuno-ELISA Chagas (Wama Diagnóstica, Brazil).

ELISA testing was carried out to determine potential antigenic cross-reactivity of four IBMP chimeric T. cruzi antigens and four commercial Chagas tests against human leishmaniasis antibodies (RI > 1.10), employing a panel of 829 serum samples consisting of 600 samples positive for American cutaneous leishmaniasis and 229 positive for visceral leishmaniasis. Of the 829 serum samples, latent class analysis indicated that 25 ACL (4.16%) and 57 VL samples (24.89%) were potentially coinfected with T. cruzi; these were subsequently excluded from the study. Cross-reactivity results for all chimeric antigens and commercial tests are illustrated in Fig. 2. ACL-positive samples returned low mean RI values (≤0.36) under all four chimeric antigens tested. Although low mean RI values were also obtained using VL-positive samples (0.38 to 0.47), these were higher than those observed in the ACL-positive samples. With respect to the commercial Chagas tests evaluated, ELISA Chagas III yielded the highest mean RI value (1.00).

As demonstrated in Fig. 2, the IBMP chimeric antigens presented very low cross-reactivity. Regarding the ACL-positive samples, the incidence of cross-reactivity ranged from 0.35% (IBMP-8.3) to 0.70% (IBMP-8.1 and IBMP-8.2), while cross-reactivity in the VL-positive samples was observed in six samples (3.49%, IBMP-8.2) and one sample (0.58%, IBMP-8.3). No cross-reactions were observed from the IBMP-8.4 antigen in either ACL or VL samples. Similarly, no cross-reactions were found when VL-positive samples were assayed with the IBMP-8.1 antigen.

In the commercial tests analyzed, with the exception of the Imuno-ELISA Chagas kit, high numbers of cross-reactions were observed. The Gold ELISA Chagas kit presented 19.30% and 20.93% of reactivity for ACL- and VL-positive samples, respectively. ELISA Chagas III had the highest incidence of cross-reactivity, as this kit misdiagnosed 315 ACL-positive samples (54.78%) as chronic Chagas disease. A high degree of cross-reactivity was also seen under indirect immunofluorescence (IIF) when testing ACL-positive samples (35.19%). Of the false-positive results returned by commercial testing (*n* = 96), 46% were correctly diagnosed using the IBMP antigens.

A combined analysis that included all ACL and VL samples with both cross-reactive and gray zone results indicated fewer unreliable events in assays using the IBMP chimeric antigens than in the commercial tests evaluated (Fig. 2). Considering all leishmaniasis-positive samples (ACL+VL), undesirable results ranged from 1.3% for IBMP-8.3 and 1.7% for IBMP-8.4 to 2.4% for IBMP-8.1 and 4.7% for IBMP-8.2, while Gold ELISA Chagas and Chagas ELISA III produced 27.7% and 58.1% undesirable results, respectively. Similar results were also observed when ACL-positive samples were used to compare IBMP-ELISA and IIF; undesirable results were returned in 35.2% of the ACL samples assayed with IIF, versus 1% with IBMP-8.3 and IBMP-8.4, 1.5% with IBMP-8.1, and 3.3% with IBMP-8.2. In contrast, the number of undesirable results was identical when assaying ACL-positive samples with Imuno-ELISA Chagas or ELISAs employing the IBMP-8.1, IBMP-8.3, or IBMP-8.4 chimeric antigens.

The expected agreement among the results returned by the IBMP chimeric antigens ranged from 97.3% for IBMP-8.2 and 99.1% for IBMP-8.1 and 99.3% for IBMP-8.3 to 100% for IBMP-8.4, demonstrating almost perfect/perfect agreement with LCA. In regard to the commercial tests, higher agreement was shown for Gold ELISA Chagas (75.8%; indicating substantial agreement), followed by ELISA Chagas III (31.0%; fair agreement). Indirect immunofluorescence testing and the Imuno-ELISA Chagas kit, assayed only with ACL-positive samples, produced substantial and almost perfect agreement, respectively, with LCA (Table 1).

**TABLE 1:** Strength of agreement of the IBMP chimeric antigens and commercial tests in the diagnosis of Trypanosoma cruzi IgG detection

Diagnostic test	Kappa value	Agreement
ELISA		
IBMP-8.1	0.991	Almost perfect
IBMP-8.2	0.973	Almost perfect
IBMP-8.3	0.993	Almost perfect
IBMP-8.4	1.000	Perfect
Gold ELISA Chagas	0.758	Substantial
ELISA Chagas III	0.310	Fair
Imuno-ELISA Chagas^*a*^	0.995	Almost perfect
IIF		
Imunocruzi^*a*^	0.755	Substantial

a Test assayed exclusively with American cutaneous leishmaniasis-positive samples.

## DISCUSSION

The laboratory diagnosis of chronic CD is complicated by the absence of a suitable reference test. Differences in the diagnostic performance of commercial kits have led the World Health Organization to recommend a combination of serological testing to reliably diagnose infection. CD diagnosis has become even more arduous due to coendemicity with other kinetoplastids. Antigen composition, used to detect specific anti-T. cruzi antibodies, and the high intraspecific genetic diversity of these parasites are the main culprits underlying these limitations. Accordingly, tests offering proven efficacy are urgently needed, and the use of chimeric T. cruzi antigenic matrices may be useful in achieving this objective. The present study employed four chimeric antigens to diagnose CD-negative samples exhibiting previously confirmed positivity for American cutaneous or visceral leishmaniasis. All assays involving the IBMP chimeric antigens exhibited weak seropositivity to leishmaniasis infection in comparison to that in the commercial immunoassays evaluated.

Cross-reactivity with anti-*Leishmania* sp. antibodies has already been reported in commercial testing designed to diagnose T. cruzi infection, mostly in tests used conventionally (13, 16, 34, 35). Sánchez et al. (36) reported that an ELISA, using total epimastigote extracts from the Ninoa and Queretaro strains as antigens, cross-reacted with 16% of sera from individuals infected with *Leishmania* spp. A Venezuelan study using epimastigotes of the DTU TcI Dm28c T. cruzi strain under direct agglutination reported seropositivity for 3 out of 9 (∼33%) sera from individuals with visceral leishmaniasis (37). Another study, employing an ELISA coated with the whole extract of T. cruzi Y strain epimastigotes, showed 92.31% and 75% cross-reactivity in panels of visceral and American cutaneous leishmaniasis-positive samples, respectively (16). These authors also demonstrated a high number of cross-reactions when using commercial ELISA kits manufactured with T. cruzi epimastigote antigens, including ELISA Chagas III (92.31% for VL and 12.5% for ACL), ELISAcruzi (bioMérieux Brazil SA; 84.6% for VL and 87.5% for ACL), and Chagatek ELISA (Laboratório Lemos, Argentina; 84.6% for VL and 87.5% for ACL). A previous investigation conducted by our group reported 42.9% and 17.1% cross-reactivity in *Leishmania* sp. samples assayed with ELISA Chagas III and Gold ELISA Chagas kits, respectively (13). It is important to emphasize that all of the above-referenced studies employed a low number of *Leishmania* sp. samples. Thus, the present investigation endeavored to use a much larger number of ACL-positive (600 samples) and VL-positive (229 samples) sera. Accordingly, the rates of cross-reactivity found in the present study were 54.78% and 18.02% for the conventionally used ELISA Chagas III test, versus 19.30% and 20.93% for Gold ELISA Chagas, considering ACL- and VL-positive samples, respectively.

The use of recombinant proteins has contributed to the development of more accurate testing for chronic CD (13, 16). While commercial kits employing recombinant proteins as antigens have demonstrated reduced cross-reactivity, false-positive results in *Leishmania* sp. samples continue to be reported (13, 16, 35). In the present study, the unconventional commercially available Imuno-Chagas ELISA yielded 0.25% false positivity in the ACL samples. It is important to note that this test uses only recombinant antigens, in contrast to Gold ELISA Chagas, which is based on both purified lysates from Brazilian strains of T. cruzi epimastigotes and recombinant proteins. The latter presented a much higher incidence of cross-reactivity (5.32%), suggesting that this complex mixture of antigens could misdiagnose individuals residing in settings characterized by T. cruzi and *Leishmania* species coendemicity. Furthermore, this incidence is in accordance with our previous findings (13) demonstrating greater cross-reactivity with *Leishmania* spp. in Gold ELISA Chagas assays compared to other commercial ELISAs employing only recombinant proteins, such as Pathozyme Chagas (Omega Diagnostics, Scotland, UK).

In contrast to the present results, some studies have reported no cross-reactions through the use of recombinant proteins. For instance, Chagastest Rec v3.0 (Wiener, Argentina) was shown to not recognize *Leishmania* sp.-specific antibodies when assayed with 13 samples positive for VL and 8 positive for ACL (16). Another study examining the use of trypomastigote small surface antigens (TSSA) found no cross-reactions in a panel of 60 *Leishmania* sp.-positive samples (38). Discrepancies in the incidence of cross-reactivity with *Leishmania* spp. could be attributable to the number of samples used. Despite the reported lower incidence of cross-reactivity in tests employing recombinant proteins, we nonetheless affirm that recombinant antigen-based tests should be used with great caution in areas where T. cruzi and *Leishmania* sp. are coendemic. Nonetheless, it is important to mention that 164 precharacterized leishmaniasis-positive samples from Spain were found to be nonreactive for T. cruzi antibodies when assayed by Elecsys Chagas, an automated electrochemiluminescence immunoassay employing soluble forms of recombinant T. cruzi antigens derived from flagellar calcium binding protein, flagellar repetitive antigen, and cruzipain (39).

The use of chimeric recombinant proteins has been recognized as a strategy to minimize or annul the potential for cross-reactions with *Leishmania* spp. in areas where T. cruzi and Leishmania spp. are coendemic (16, 19). The four chimeric proteins employed here, composed of in-tandem repetitive and conserved amino acid sequences from several T. cruzi proteins, were previously evaluated to diagnose chronic CD in settings where it is endemic and nonendemic and achieved accuracy rates above 96% for IBMP-8.2 and over 98% for the other three chimeric antigens (20). Our previously reported rates of cross-reactivity were 1.31% in 153 *Leishmania* sp.-positive samples assayed with IBMP-8.1, and 0.65% for those assayed by IBMP-8.3 and IBMP-8.4. The IBMP-8.2 chimera exhibited no cross-reactivity. These findings were consistent with another previous study utilizing liquid microarray analysis, which also detected no cross-reactions in the 18 *Leishmania* sp.-positive samples evaluated (25). All of these studies were mainly limited by the small number of quantitative samples assayed, the absence of clinical characterization regarding cutaneous or visceral leishmaniasis forms, and biased selection of two commercial tests employed to diagnose *Leishmania* sp.-positive samples as negative for T. cruzi infection. Therefore, the present study applied latent class analysis to diagnose chronic CD in a well-characterized set consisting of 600 ACL and 229 VL-positive samples, similarly to a previous study conducted by our group employing LCA to classify samples as T. cruzi positive or negative based on a statistically well-established response pattern (32). Accordingly, 25 ACL (4.16%) and 57 VL (24.9%) samples were excluded due to being classified under LCA as coinfected with T. cruzi. In contrast, if two commercial tests had been used to identify coinfection with T. cruzi in the present panel, the resulting combination of testing by Gold ELISA Chagas and ELISA Chagas III would result in 420 positive or discordant ACL (70%) and 133 VL (58.1%) samples being excluded from the present cross-reaction investigation. Through the use of LCA, no or negligible cross-reactivity was demonstrated for all four chimeric antigens in both ACL and VL samples, with the exception of IBMP-8.2 (3.49% of seropositivity in VL). Global agreement analysis indicated more than 97% concordance for IBMP-8.2 and 99% for IBMP-8.1 and IBMP-8.3 chimeric antigens. Interestingly, perfect agreement was seen with respect to IBMP-8.4.

To the best of our knowledge, no other investigations have attempted to assay a similarly large sample of sera with well-defined leishmaniasis diagnosis, with results demonstrating the lack of potential for cross-reactivity with T. cruzi antigens. Moreover, our findings indicate that the use of chimeric antigens in areas where *Leishmania* and T. cruzi are coendemic would ultimately reduce diagnostic costs due to a significant reduction in the number of samples requiring reassaying. Thus, we suggest that the use of IBMP chimeric antigens, especially IBMP-8.4, can be safely applied in the diagnosis of Chagas disease in settings where *Leishmania* and T. cruzi are considered coendemic.
